# Malignant Myoepithelioma of the Head and Neck: Demographics, Clinicopathological Characteristics, Treatment, and Prognosis

**DOI:** 10.3389/fonc.2022.754967

**Published:** 2022-06-30

**Authors:** Jia-Qi Wang, Rong-Xin Deng, Hui Liu, Yuan Luo, Meng-Meng Lu, Zhi-Cheng Yang

**Affiliations:** ^1^Department of Oral and Maxillofacial Surgery, Shanghai Stomatological Hospital & School of Stomatology, Fudan University, Shanghai, China; ^2^Shanghai Key Laboratory of Craniomaxillofacial Development and Diseases, Fudan University, Shanghai, China

**Keywords:** malignant myoepithelioma, head and neck cancer, SEER database, nomogram, overall survival

## Abstract

Malignant myoepithelioma of the head and neck (HNMM) is a rare malignancy, and its characteristics and survival rates have not been well-defined. This study aimed to define the epidemiology of HNMM and identify the prognostic factors associated with the disease. Data on all patients diagnosed with HNMM between 1991 and 2016 were gathered from the Surveillance Epidemiology and End Results (SEER) database. The demographics, clinicopathological characteristics, treatment, and prognoses of the patients were described. Cox regression analysis was used to identify the prognostic factors, and the prognostic nomograms for overall survival (OS) and disease-specific survival (DSS) were constructed. A total of 333 cases of HNMM were identified. The average age at diagnosis was 60.6 years, and 50.1% of the patients were men. After diagnosis, 46.2% of patients underwent surgery alone, 43.5% of patients underwent surgery and radiotherapy, and 3.6% of patients received only radiotherapy. Survival analysis showed that the 5-year OS and DSS for all HNMM patients were 69.7 and 82.1%, respectively. In the multivariate analysis model, the undifferentiated pathological grade (P <0.05) and M1 in the M category (P <0.01) were independent prognostic factors for poor OS and DSS, whereas the use of surgical resection was an independent favorable prognostic factor for both OS and DSS (P <0.05). The prognostic nomograms for OS and DSS prediction were constructed; the C-index values for OS and DSS prediction were 0.78 (95% CI 0.70–0.86) and 0.79 (95% CI 0.67–0.90), respectively. In conclusion, this SEER data-based study demonstrated that HNMM patients often had a favorable prognosis, and distant metastasis, pathological grade, and the use of surgery contributed to their survival. Furthermore, we developed a prognostic nomogram to predict OS and DSS for HNMM patients to aid physicians in the clinical management of this rare disease.

## Introduction

A myoepithelial tumor is a rare malignancy that is composed almost exclusively of cells with myoepithelial differentiation. Myoepithelial tumors were classified among salivary gland tumors as separate entities by the World Health Organization in 1991 (1) . These can be can categorized as benign and malignant myoepitheliomas. Malignant myoepithelioma (MM) is a neoplasm that exhibits a wide morphological and cytological diversity similar to its benign counterpart, myoepithelioma, with evidence of malignant change. Due to a lack of specific symptoms or imaging characteristics, it is impossible to differentiate benign from malignant myoepitheliomas based on clinical information. Therefore, a biopsy is required for the diagnosis of this disease. Malignant myoepitheliomas often present with an infiltrative growth pattern, angiolymphatic or perineural invasion, and a propensity for metastasis and recurrence (2–4). Nagao et al. (5) reported that myoepithelial tumors with high cell proliferative activity suggest malignancy, irrespective of their histological appearance.

Malignant myoepitheliomas are often located in the salivary glands (6–8). In addition to the salivary gland, previous studies have reported that this disease may arise in other head and neck locations such as the nasal cavity, nasopharynx, and the maxillary sinus (9–14). Owing to its rarity, much of the current knowledge and clinical approaches to malignant myoepithelioma of the head and neck (HNMM) are limited to generalizations from malignant myoepitheliomas located in other anatomical regions (7). Furthermore, there is a lack of population data, and no studies so far have defined the clinicopathological characteristics and determined the factors influencing survival in a large cohort; these factors limit the understanding of this rare disease. Thus, we conducted the present study to describe the demographics, clinicopathologic characteristics, treatment regimen, and prognosis of HNMM patients using data from the Surveillance Epidemiology and End Results (SEER) database.

## Materials and Methods

### Participants

A population-based search for patients diagnosed with HNMM between 1991 and 2016 was carried out in the SEER database of “SEER 18 Regs Custom Data with additional treatment fields, Nov 2018 Sub (1975–2016)” using SEER*STAT 8.3.9 software. Given that SEER is a publicly available database, institutional review board approval was not required (Ethics committee of Shanghai Stomatological Hospital). The International Classification of Diseases for Oncology (ICD-O) topography code 8982/3 was used to identify all HNMM patients. The study variables included demographic information, clinicopathological factors, treatment, and prognosis. Specific information retrieved included data on age at diagnosis, sex, race, tumor grade, anatomical site, TNM stage (AJCC 7th edition), surgery, radiotherapy, survival status, and survival time (overall survival, OS; disease-specific survival, DSS). OS was defined as the interval from initial diagnosis to death from any cause or last follow-up, and DSS was defined as the interval from initial diagnosis to death caused by this disease.

### Statistical Analysis

Descriptive statistics were calculated for all demographic and clinicopathological characteristics. Survival analyses for OS and DSS were performed using the Kaplan–Meier curve and log-rank tests. Univariate and multivariate Cox regression analyses were used to assess the predictive performance of each covariate for OS and DSS. All survival analyses were performed using MedCalc software (version 15.2.2, Mariakerke, Belgium), and the prognostic nomograms for OS and DSS predictions were constructed using R version 3.6.0 (R Foundation for Statistical Computing, Vienna, Austria). P <0.05 was considered statistically significant.

## Results

A total of 333 patients diagnosed with HNMM between 1991 and 2016 were found in the SEER database. Patient characteristics are shown in **Table 1**. Of these patients, 50.1% were women, and 72.7% were white. The average and median age at diagnosis were 60.6 and 63 years, respectively (range: 1–94 years). The salivary gland was the most affected site, followed by the oral cavity. Definitive staging was available in 67.3% of cases, with almost equal distributions at each stage (stage I, 21.9%; stage II, 25.8%; stage III, 25.0%; stage IV, 27.3%). Among 243 (OS) and 245 (DSS) patients with definitive information on metastases, lymph node and distant metastases were observed in 37/243 patients and 16/245 patients, respectively. As for the treatment regimen, 46.2% of patients underwent surgery alone, 43.6% of patients received surgery and radiotherapy, 3.6% of patients received radiotherapy alone, and 6.3% received neither. Compared with those receiving surgery plus radiotherapy, HNMM patients receiving surgery alone tended to exhibit well differentiated, early-stage tumors (TNM-I/II, T1/T2, lymph node-negative tumors) (**Supplementary Table 1**). Moreover, patients who could not receive surgery were more likely to have exhibited distant metastases (6/33 vs. 10/299).

**Table 1:** Patients’ characteristics.

Characteristics	Total (N = 333)
Age (Year)	
Mean	60.6
Median	63
Min	1
Max	94
Sex	
Female	167 (50.1%)
Male	166 (49.9%)
Race	
White	242 (72.7%)
Black	54 (15.2%)
Other (American Indian/AK Native, Asian/Pacific Islander)	35 (10.5%)
Unknown	2 (0.6%)
Tumor Grade	
Well	42 (23.5%)
Moderately	77 (42.0%)
Poorly	34 (17.6%)
Undifferentiated	34 (16.9%)
Unknown	146
Primary Site	
Salivary Gland	245 (76.0%)
Oral Cavity	52 (13.5%)
Nasal cavity & accessory sinuses	21 (1.1%)
Pharynx & Larynx	10 (9.4%)
Other	5
TNM	
I	50
II	59
III	57
IV	58
Unknown	109
T category	
T1	52 (16.2%)
T2	66 (33.5%)
T3	67 (32.9%)
T4	45 (23.3%)
Unknown	103
N category	
N0	206 (85.6%)
N1	17 (12.8%)
N2	20 (6.6%)
Unknown	90
M category	
M0	229 (92.3%)
M1	16 (7.7%)
Unknown	88
Surgery	
Yes	299 (89.8%)
No	33
Unknown	1
Radiotherapy	
Yes	157
No	176
Treatment modality	
Surgery + radiotherapy	145 (43.6%)
Surgery Alone	154 (46.2%)
Radiotherapy Alone	12 (3.6%)
None	21 (6.3%)
Unknown	1(0.3%)

**Figure 1:**
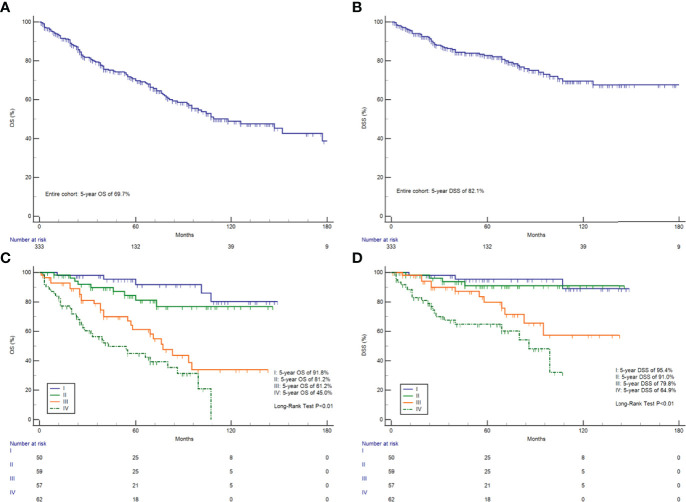
Survival analysis. OS **(A)** and DSS **(B)** in all 333 HNMM patients; OS **(C)** and DSS **(D)** analysis stratified by AJCC-TNM staging.

**Figure 2:**
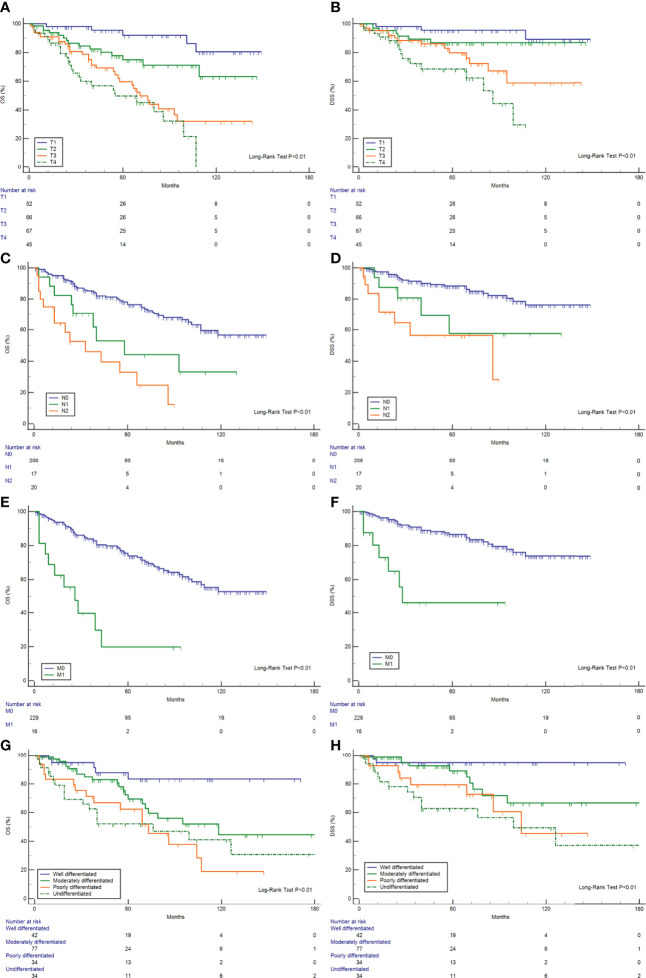
OS and DSS analysis. **(A)** OS and T category; **(B)** OS and N category; **(C)** OS and M category; **(D)** OS and pathological grade; **(E)** DSS and T category; **(F)** DSS and N category; **(G)** DSS and M category; and **(H)** DSS and pathological grade.

The Kaplan–Meier curves for OS and DSS showed that the 5-year OS and DSS in the entire cohort were 69.7 and 82.1%, respectively (**Figures 1A, B**). The median OS was 118 months (95% CI, 93–177). Survival analysis revealed a statistically significant difference in OS and DSS stratified according to the stage at presentation (P <0.01) (**Figures 1C, D**). Similarly, pathological grade and T/N/M categories were significantly associated with both OS and DSS (**Figure 2**). Male sex and the use of radiotherapy were associated with worse DSS (P = 0.04 for both). Younger age was associated with significantly better OS (P <0.01). However, race and primary site were not significantly associated with OS and DSS.

**Figure 3:**
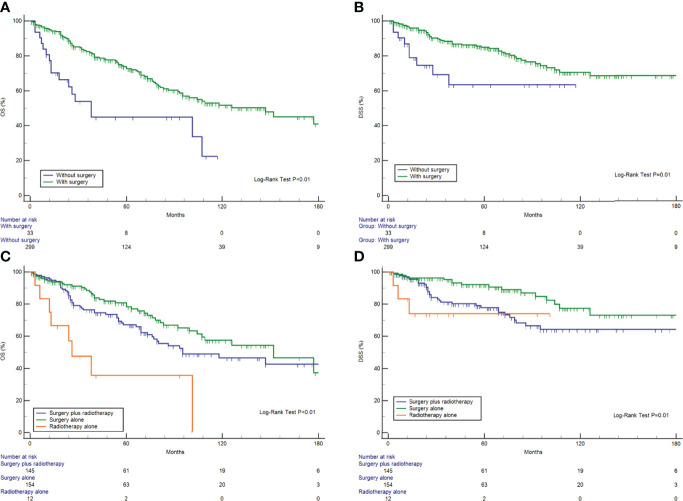
Survival analysis stratified by surgery **(A**, **B)** and treatment modalities **(C**, **D)**.

As for treatment modality, surgical resection was associated with better DSS and OS (**Figures 3A, B**). A Kaplan–Meier analysis was used to compare the relative survival curves for HNMM patients receiving surgical resection, radiotherapy, both, or neither (**Figures 3C, D**). Differences in OS were observed between the patients treated with surgery alone and radiotherapy alone (P = 0.01), whereas differences in DSS were observed between those treated with bimodal therapy and surgical resection (P = 0.01) (**Supplementary Table 2**).

We then further compared the efficacy of treatment modalities stratified by tumor stage and the presence of lymph node metastases. Surgery plus radiotherapy could not significantly improve OS or DSS, compared with surgery alone, among patients with late-stage (III/IV) or lymph node metastasis (P >0.05). Similarly, no significant differences were observed between treatment modalities (surgery alone vs. surgery plus radiation) among early-stage patients (I/II) (P >0.05) (**Supplementary Figure 1**).

**Tables 2**, **3** show the results of the univariate and multivariate Cox regression analyses for OS and DSS, respectively. In the multivariate analysis model, an absence of differentiation in terms of pathological grade (OS: HR = 5.46, 95% CI 1.62–18.4, P <0.01; DSS: HR = 8.20, 95% CI 1.31–51.4, P = 0.03) and M1 in the M category (OS: HR = 9.98, 95% CI 3.57–27, P <0.01; DSS: HR = 18.6, 95% CI 4.67–74.3, P <0.01) were independent prognostic factors for worse OS and DSS, while the use of surgical resection was an independent favorable prognostic indicator for both OS and DSS (OS: HR = 0.15, 95% CI 0.05–0.47 P <0.01; DSS: HR = 0.14, 95% CI 0.02–0.83, P = 0.03). Additionally, N2 in the N category was an independent, unfavorable, prognostic factor for OS (HR = 3.20, 95% CI 1.31–7.80, P = 0.01).

**Table 2:** Univariate COX regression analysis for OS and DSS.

Characteristic	OS		DSS	
	HR with 95% CI	P-value	HR with 95% CI	P-value
Age (≥61 vs <61)	2.11 (1.42–3.15)	<0.01	1.03 (0.62–1.71)	0.92
Sex (Male vs Female)	1.41 (0.97–2.06)	0.07	1.69 (1.01–2.84)	0.04
Race				
White	Reference		Reference	
Black	0.77 (0.46–1.29)	0.31	0.75 (0.37–1.54)	0.44
Other	0.54 (0.27–1.13)	0.10	0.51 (0.18–1.42)	0.20
Grade				
Well	Reference		Reference	
Moderately	2.72 (1.02–7.21)	0.04	3.17 (0.69–14.5)	0.14
Poorly	5.27 (1.93–14.4)	<0.01	6.46 (1.37–30.4)	0.02
Undifferentiated	4.98 (1.85–13.4)	<0.01	9.77 (2.22–43.1)	<0.01
Primary site				
Salivary Gland	Reference		Reference	
Oral Cavity	1.01 (0.59–1.73)	0.98	1.60 (0.84–3.07)	0.16
Nasal cavity & accessory sinuses	1.11 (0.56–2.21)	0.77	1.89 (0.84–4.25)	0.12
Pharynx & Larynx	2.84 (0.99–7.81)	0.06	2.80 (0.67–11.7)	0.16
Other	1.45 (0.46–4.61)	0.53	2.29 (0.55–9.52)	0.26
T category				
T1	Reference		Reference	
T2	3.02 (1.10–8.31)	0.03	2.29 (0.59–8.87)	0.23
T3	6.62 (2.57–17.1)	<0.01	4.85 (1.39–16.9)	0.0132
T4	9.29 (3.53–24.4)	<0.01	9.17 (2.64–31.9)	<0.01
N category				
N0	Reference		Reference	
N1	2.45 (1.21–4.97)	0.01	2.60 (1.01–6.75)	0.04
N2	5.13 (2.83–9.31)	<0.01	5.56 (2.51–12.3)	<0.01
M category (M0 vs M1)	4.82 (2.53–9.20)	<0.01	5.65 (2.48–12.9)	<0.01
Surgery (Yes vs No)	0.39 (0.23–0.66)	<0.01	0.42 (0.21–0.85)	0.016
Radiation (Yes vs No)	1.34 (0.92–1.94)	0.13	1.69 (1.01–2.83)	0.046

**Table 3:** Multivariate COX regression analysis for OS and DSS.

Characteristic	OS		DSS	
	HR with 95% CI	P-value	HR with 95% CI	P-value
Age (≥61 vs <61)	1.35 (0.67–2.70)	0.40	N/A	N/A
Sex (Male vs Female)	N/A	N/A	1.63 (0.53–5.02)	0.40
Grade				
Well	Reference		Reference	
Moderately	3.22 (0.99–10.5)	0.06	4.31 (0.66–28.4)	0.13
Poorly	3.12 (0.88–11.0)	0.08	3.05 (0.42–22.0)	0.27
Undifferentiated	5.46 (1.62–18.4)	<0.01	8.20 (1.31–51.4)	0.03
T category				
T1	Reference		Reference	
T2	2.97 (0.80–11.0)	0.10	1.30 (0.21–8.06)	0.77
T3	2.43 (0.62–9.50)	0.20	1.79 (0.31–10.5)	0.52
T4	3.22 (0.82–12.7)	0.09	2.84 (0.47–17.3)	0.26
N category				
N0	Reference		Reference	
N1	1.02 (0.30–3.44)	0.97	1.01 (0.18–5.65)	0.99
N2	3.20 (1.31–7.80)	0.01	3.36 (0.99–11.4)	0.06
M category (M0 vs M1)	9.98 (3.57–27.9)	<0.01	18.6 (4.67–74.3)	<0.01
Surgery (Yes vs No)	0.15 (0.05–0.47)	<0.01	0.14 (0.02–0.83)	0.03
Radiation (Yes vs No)	N/A	N/A	1.11 (0.40–3.03)	0.84

Only significant factors from univariate Cox analysis were included in multivariate Cox analysis. N/A, not available.

**Figure 4:**
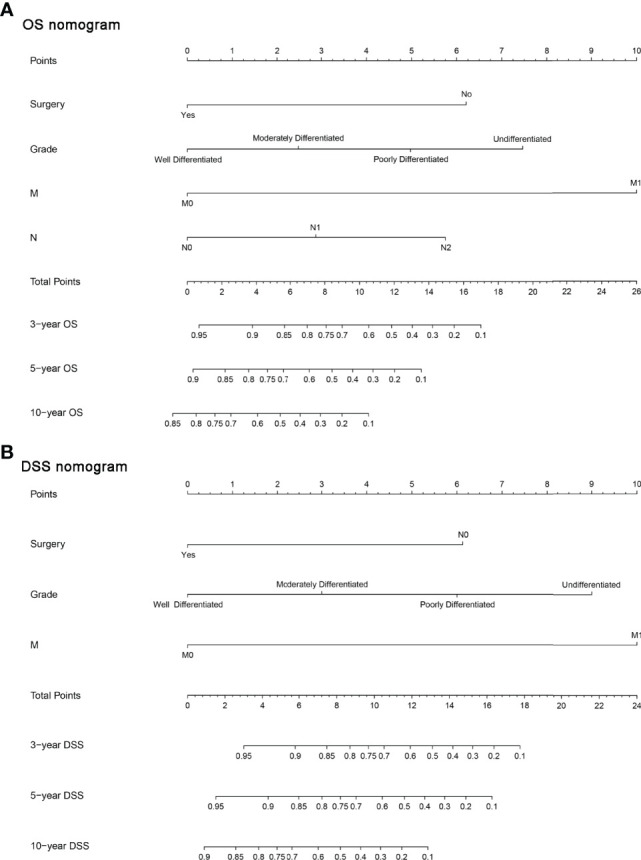
The constructed prognostic nomogram for OS **(A)** and DSS **(B)** prediction. Each variable was assigned a score on the point scale. By summing the total score and locating it on the total point scale, a straight line was drawn down to determine the estimated probability of OS and DSS.

**Figure 5:**
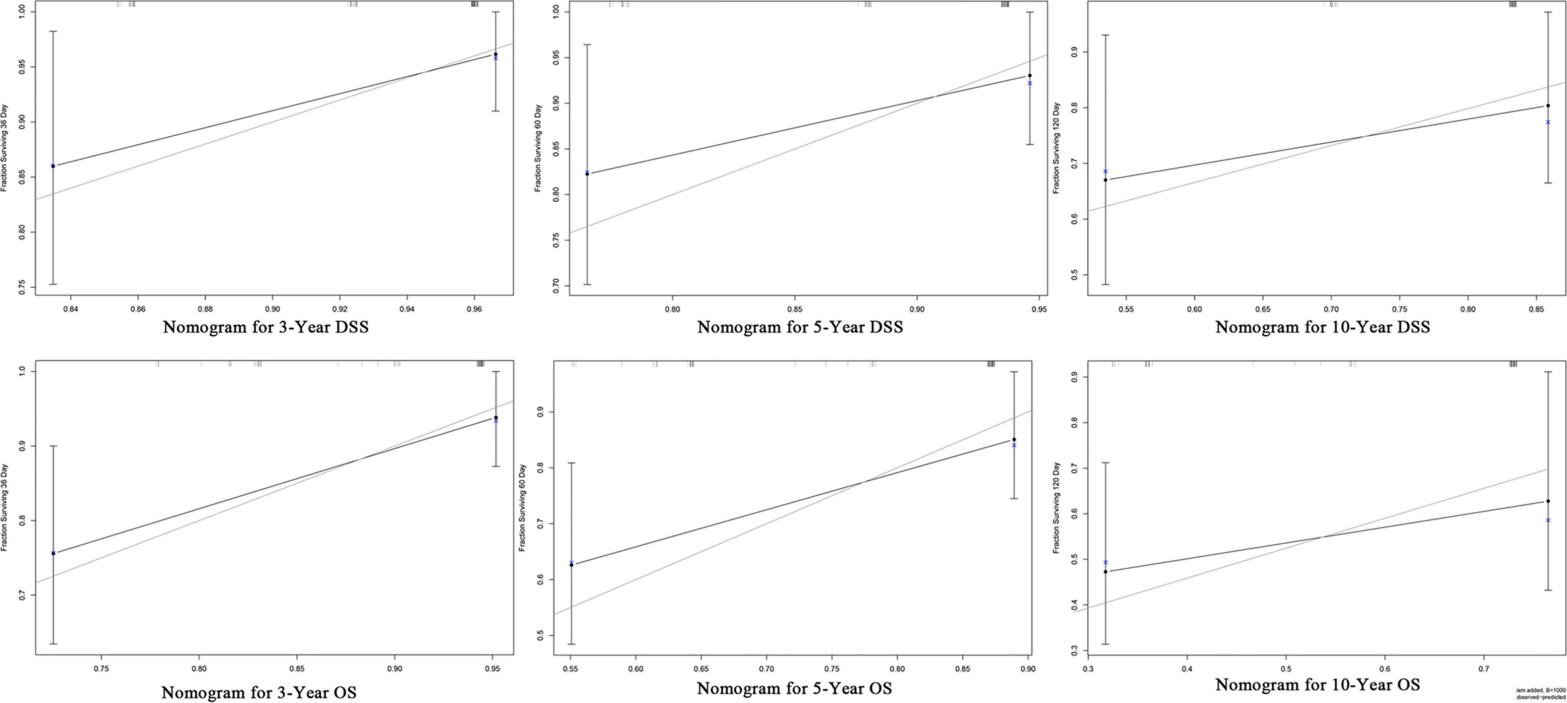
Calibration curves for the 3-, 5-, and 10-year DSS/OS.

Furthermore, we constructed the prognostic nomograms for OS and DSS among HNMM patients using independent prognostic factors from multivariate Cox regression analysis. As shown in **Figure 4**, distant metastasis contributed the most to both OS and DSS, followed by pathological grade and the use of surgery. The C-index values for OS and DSS predictions were 0.78 (95% CI 0.70–0.86) and 0.79 (95% CI 0.67–0.90), respectively. The 3-, 5-, and 10-year calibration curves showed excellent agreement between the predicted and observed values (**Figure 5**).

## Discussion

Data on HNMM are relatively limited. In addition, there is a demand for large-scale cohort studies to determine the clinicopathological determinants of survival and treatment modalities for this rare malignancy. This study, using data from the SEER database, permitted the analysis of treatment and outcomes using population-based data relating to this rare malignancy. This study is, to our knowledge, based on the largest cohort of HNMM patients in its description of demographics and clinicopathological characteristics as well as its definition of prognostic factors.

Demographically, our results concerning age agreed with data previously reported in the literature related to HNMM, with the peak incidence recorded in the sixth decade of life (range: 14–96 years) (11, 15–17). Although age was significantly associated with OS rather than DSS, this significant association disappeared after adjusting for other variables in the multivariate Cox regression analysis. The sex distribution results contradicted those of the previously reported studies. Nagao et al. reported a predominance of women over men (2:1) among 10 patients with MM of the salivary gland, whereas Yu et al. observed a predominance of men (1.7:1) (5, 18). However, our cohort found an equal sex distribution, with 167 women and 166 men. One possible explanation for this discrepancy is that several previous studies only focused on patients with MM of the salivary gland. In our study, we included patients with MM in other head and neck regions, not only in the salivary gland. Notably, this study is the first to suggest that male patients with HNMM have a worse prognosis than their female counterparts. Race appeared to have no statistically significant effect on the survival of HNMM patients. This finding is inconsistent with previous reports that indicate that race is an independent prognostic factor in other head and neck malignancies (19, 20).

In terms of clinicopathology, most tumors (76%) occurred in the salivary glands in this cohort; this result is consistent with the results of previous studies (17). A survival analysis stratified in terms of the primary sites showed no significant differences in DSS and OS. Patients with MM of the salivary gland had a survival rate similar to those with MM of other parts of the head and neck. This result suggests that these patients may belong to one entity. Previous studies have revealed that pathological grade is an important prognostic reference for tumors in the head and neck region (21–24). This study also indicated a significant association between pathological differentiation and survival, and multivariate analysis demonstrated that pathological differentiation was independently associated with OS and DSS. In the constructed nomograms, pathological differentiation had the second highest contribution to OS and DSS predictions. This finding demonstrates the importance of the pathological differentiation of MM located in the head and neck region on prognosis; thus, physicians should evaluate the prognosis in terms of pathological differentiation. AJCC-TNM staging plays an essential role in treatment planning and prognosis evaluation. Based on the available information, in this cohort, an equal distribution of the AJCC-TNM stage was observed among the 234 patients. Meanwhile, 19.4 and 7.7% of patients had lymph node and distant metastases, respectively, signifying the aggressive nature of this rare malignancy *via* hematogenous and lymphatic spread. Both the N and M categories were independently associated with OS and DSS. For example, patients with distant metastases have a dismal prognosis (OS, 26 months; DSS, 28 months). Therefore, an early examination and diagnosis is vital to improve survival and decrease the possibility of metastases.

Overall, the prognosis of HNMM patients is better than that of patients with other malignancies in the head and neck region (25, 26). According to the largest case series reported to date, the 5-year cumulative survival rate of 59 Chinese patients with HNMM was 62% (27). In this cohort, our data showed a 5-year OS rate of 69.7%. One possible explanation for this difference is the higher proportion of stage III (23/59 cases) and stage IV (19/59) patients in the cohort in the study by Zhao etal. (27) than in this study. Furthermore, our data revealed that a significant majority of cases (90.1%) were treated with surgical resection, and the use of surgery was an independent favorable prognostic factor. Surgery may decrease the risk of death from all causes and HNMM by 85 and 86%, respectively. Surgery significantly prolonged OS by approximately 109 months (147 months vs. 38 months). Therefore, surgery is the optimal treatment strategy for patients with HNMM. A previous study has also demonstrated that surgical resection is the preferred treatment for HNMM. However, the requirements for a first surgery are high. Furthermore, if the resection is not complete, it is easy for relapse to occur, and the operation and adjuvant treatment often do not deliver satisfactory results (27). Radiotherapy is an alternative regimen for patients who cannot tolerate surgery; it can also serve as an adjuvant treatment for patients undergoing surgery. In this cohort, approximately half of all patients (47.1%) received radiotherapy. The addition of radiotherapy to surgery did not significantly prolong the OS or DSS. Moreover, patients receiving a combination of surgery and radiotherapy had a considerably shorter duration of DSS than those who underwent surgery alone. This result may be attributed to the fact that patients receiving radiotherapy plus surgery mostly had advanced-stage tumors and that patients with radiotherapy were more likely to have late-stage tumors (76/115 vs. 43/113). Radiotherapy alone is sometimes used palliatively, as was the case in 3.6% of patients in our cohort who received radiotherapy alone. Moreover, our data showed that patients who underwent surgery alone had a substantially longer OS than those who underwent radiotherapy alone, suggesting that radiotherapy could not replace surgery among patients with HNMM. However, only 12 out of 333 patients received radiotherapy alone. Hence, it is difficult to arrive at a solid conclusion on the role of radiotherapy in HNMM owing to the small sample size. Moreover, the efficacy of chemotherapy for HNMM could not be evaluated because of insufficient information. Therefore, the optimal treatment regime for HNMM patients still needs to be confirmed in future research.

Despite a large sample size for this rare malignancy, this study has several limitations. First, certain variables could not be precisely analyzed retrospectively, including tumor recurrence and comorbidities. Second, no information on chemotherapy was available in the SEER database; thus, the analysis was limited in terms of exploring the optimal regimen for this malignancy. The lack of data on cancer control and tumor recurrence in the SEER database restricted the potential knowledge we could have gained concerning this rare disease. Third, concerns emerged regarding the misclassification and lack of clinicopathologic variables, particularly tumor grade and histological differentiation, in the database. For example, there was no information on the TNM stage for 109 patients and on the pathological grade for 146 patients.

In conclusion, HNMM is a rare malignancy that often occurs in the sixth decade of life with an equal sex distribution. It has a relatively good survival rate with a 5-year OS and DSS of 69.7 and 82.1%, respectively. Pathological grade and distant metastasis are independently associated with its prognosis. Surgical resection confers OS and DSS benefits in patients with HNMM. Furthermore, distant metastasis, pathological grade, and the use of surgery contribute to the establishment of prognostic predictions of OS and DSS among HNMM patients.

## Data Availability Statement

The original contributions presented in the study are included in the article/**Supplementary Material**. Further inquiries can be directed to the corresponding author.

## Author Contributions

J-QW: Study design, data collection, data analysis, and writing—original draft preparation. R-XD: Date collection, data analysis, and writing—original draft preparation. HL: Data analysis, software, and writing—reviewing. YL: Data collection and data analysis. M-ML: Writing—reviewing and editing. Z-CY: Study design, supervision, writing—reviewing and editing. All authors listed have made a substantial, direct, and intellectual contribution to the work and approved it for publication.

## Conflict of Interest

The authors declare that the research was conducted in the absence of any commercial or financial relationships that could be construed as a potential conflict of interest.

## Publisher’s Note

All claims expressed in this article are solely those of the authors and do not necessarily represent those of their affiliated organizations, or those of the publisher, the editors and the reviewers. Any product that may be evaluated in this article, or claim that may be made by its manufacturer, is not guaranteed or endorsed by the publisher.

## Supplementary Material

Supplementary Figure 1 Survival analysis stratified by treatment modalities (surgery plus radiation vs surgery alone) **(A**: TNM-I/II, OS; **(B**: TNM-I/II, DSS; **(C**: TNM-III/IV, OS; **(D**: TNM-III/IV, DSS; **(E**: N1+N2, OS; **(F**: N1+N2, DSS; **(G**: N0, OS; **(H**: N0, DSS).

## References

[1] Seifert G Sobin LH. Myoepithelioma. World Health Organization International Histological Classification of Tumours. In: Histological Typing of Salivary Gland Tumours, 2nd ed. Berlin, Germany: Springer-Verlag (1991). p. 20–1.

[2] Ogawa I Nishida T Miyauchi M Sato S Takata T. Dedifferentiated Malignant Myoepithelioma of the Parotid Gland. Pathol Int (2003) 53:704–9. doi: 10.1046/j.1440-1827.2003.01536.x 14516322

[3] Kong M Drill EN Morris L West L Klimstra D Gonen M. Prognostic Factors in Myoepithelial Carcinoma of Salivary Glands: A Clinicopathologic Study of 48 Cases. Am J Surg Pathol (2015) 39:931–8. doi: 10.1097/PAS.0000000000000452 PMC4939272 25970687

[4] Vilar-Gonzalez S Bradley K Rico-Perez J Vogiatzis P Golka D Nigam A. Salivary Gland Myoepithelial Carcinoma. Clin Transl Oncol (2015) 17:847–55. doi: 10.1007/s12094-015-1329-4 26133522

[5] Nagao T Sugano I Ishida Y Tajima Y Matsuzaki O Konno A. Salivary Gland Malignant Myoepithelioma: A Clinicopathologic and Immunohistochemical Study of Ten Cases. Cancer (1998) 83:1292–9. doi: 10.1002/(SICI)1097-0142(19981001)83:7<1292::AID-CNCR4>3.0.CO;2-L 9762928

[6] Vazquez A Patel TD D'aguillo CM Abdou RY Farver W Baredes S. Epithelial-Myoepithelial Carcinoma of the Salivary Glands: An Analysis of 246 Cases. Otolaryngol. Head Neck Surg (2015) 153:569–74. doi: 10.1177/0194599815594788 26195572

[7] Rastrelli M Del Fiore P Damiani GB Mocellin S Tropea S Spina R. Myoepithelioma of the Soft Tissue: A Systematic Review of Clinical Reports. Eur J Surg Oncol (2019) 45:1520–6. doi: 10.1016/j.ejso.2019.05.003 31085025

[8] Trevino M Moorthy C Kafchinski L Bustamante D. Foot Plantar Soft Tissue Malignant Myoepithelioma Tumor: Case Report and Review of the Literature. Clin Imaging (2020) 61:90–4. doi: 10.1016/j.clinimag.2019.11.014 32000118

[9] Graadt Van Roggen JF Baatenberg-De Jong RJ Verschuur HP Balhuizen JC Slootweg PJ Van Krieken JH. Myoepithelial Carcinoma (Malignant Myoepithelioma): First Report of an Occurrence in the Maxillary Sinus. Histopathology (1998) 32:239–41. doi: 10.1046/j.1365-2559.1998.00382.x 9568509

[10] Zhou SH Ruan LX Gong L Gong SQ. Primary Malignant Myoepithelioma of the Left Maxillary Sinus: A Case Report. J Int Med Res (2008) 36:362–5. 10.1177/147323000803600221 18380949

[11] Hata M Tokuuye K Shioyama Y Nomoto S Inadome Y Fukumitsu N. Malignant Myoepithelioma in the Maxillary Sinus: Case Report and Review of the Literature. Anticancer Res (2009) 29:497–501. 19331194

[12] Petersson F Chao SS Ng SB. Anaplastic Myoepithelial Carcinoma of the Sinonasal Tract: An Underrecognized Salivary-Type Tumor Among the Sinonasal Small Round Blue Cell Malignancies? Report of One Case and a Review of the Literature. Head Neck Pathol (2011) 5:144–53. doi: 10.1007/s12105-010-0226-y PMC3098327 21104210

[13] Chen L Fu Y Wang H Yang F Liu J Xia F. Myoepithelial Carcinoma of the Nasopharynx: Rare Case Report With Clinicopathologic and Immunohistochemical Features Review of Literature. Head Neck (2018) 40:E62–7. doi: 10.1002/hed.25150 29573095

[14] Silveira HA Almeida LY Nonaka CFW Alves PM Ribeiro-Silva A Leon JE. Myoepithelial Carcinoma With Rhabdoid Features in the Maxillary Sinus: Immunohistochemical and *in Situ* Hybridization Analysis of a Rare Case. Oral Oncol (2019) 93:116–9. doi: 10.1016/j.oraloncology.2019.04.015 31053364

[15] Wang J Wu Q Sun K Beng C. Quantitative Multivariate Analysis of Myoepithelioma and Myoepithelial Carcinoma. Int J Oral Maxillofac Surg (1995) 24:153–7. doi: 10.1016/S0901-5027(06)80091-3 7608581

[16] Neto AG Pineda-Daboin K Luna MA. Myoepithelioma of the Soft Tissue of the Head and Neck: A Case Report and Review of the Literature. Head Neck (2004) 26:470–3. doi: 10.1002/hed.20044 15122665

[17] Li CQ Guo ZM Liu WW Zhang Q Yang AK Yang L. [Clinical Analysis of Myoepithelial Carcinoma of Head and Neck]. Zhonghua Er Bi Yan Hou Tou Jing Wai Ke Za Zhi (2010) 45:124–7. 20398508

[18] Yu G Ma D Sun K Li T Zhang Y. Myoepithelial Carcinoma of the Salivary Glands: Behavior and Management. Chin Med J (Engl) (2003) 116:163–5. 12775221

[19] Mahal BA Inverso G Aizer AA Bruce Donoff R Chuang SK. Impact of African-American Race on Presentation, Treatment, and Survival of Head and Neck Cancer. Oral Oncol (2014) 50:1177–81. doi: 10.1016/j.oraloncology.2014.09.004 25261298

[20] Zandberg DP Liu S Goloubeva O Ord R Strome SE Suntharalingam M. Oropharyngeal Cancer as a Driver of Racial Outcome Disparities in Squamous Cell Carcinoma of the Head and Neck: 10-Year Experience at the University of Maryland Greenebaum Cancer Center. Head Neck (2016) 38:564–72. doi: 10.1002/hed.23933 PMC4461547 25488341

[21] Bjorndal K Krogdahl A Therkildsen MH Overgaard J Johansen J Kristensen CA. Salivary Gland Carcinoma in Denmark 1990-2005: A National Study of Incidence, Site and Histology. Results of the Danish Head and Neck Cancer Group (DAHANCA). Oral Oncol (2011) 47:677–82. doi: 10.1016/j.oraloncology.2011.04.020 21612974

[22] Schwarz S Zenk J Muller M Ettl T Wunsch PH Hartmann A. The Many Faces of Acinic Cell Carcinomas of the Salivary Glands: A Study of 40 Cases Relating Histological and Immunohistological Subtypes to Clinical Parameters and Prognosis. Histopathology (2012) 61:395–408. doi: 10.1111/j.1365-2559.2012.04233.x 22551398

[23] Wang YL Zhu YX Chen TZ Wang Y Sun GH Zhang L. Clinicopathologic Study of 1176 Salivary Gland Tumors in a Chinese Population: Experience of One Cancer Center 1997-2007. Acta Otolaryngol (2012) 132:879–86. doi: 10.3109/00016489.2012.662715 PMC3433083 22497626

[24] Yibulayin F Feng L Wang M Lu MM Luo Y Liu H. Head & Neck Acinar Cell Carcinoma: A Population-Based Study Using the Seer Registry. BMC Cancer (2020) 20:631. doi: 10.1186/s12885-020-07066-y 32641007 PMC7346396

[25] Yu CX Yibulayin F Feng L Wang M Lu MM Luo Y. Clinicopathological Characteristics, Treatment and Prognosis of Head & Neck Small Cell Carcinoma: A SEER Population-Based Study. BMC Cancer (2020) 20:1208. doi: 10.1186/s12885-020-07522-9 33287756 PMC7722424

[26] Yan O Xie W Teng H Fu S Chen Y Liu F. Nomograms Forecasting Long-Term Overall and Cancer Specific Survival of Patients With Head and Neck Neuroendocrine Carcinoma. Front Oncol (2021) 11:619599. doi: 10.3389/fonc.2021.619599 33659217 PMC7917297

[27] Zhao DL Wang LZ Cao H Sang JZ Gao L Cao XD. [Clinical Characteristics and Treatment of Myoepithelial Carcinoma of Head and Neck]. Lin Chung Er Bi Yan Hou Tou Jing Wai Ke Za Zhi (2019) 33:1085–8. 10.13201/j.issn.1001-1781.2019.11.019 31914301

