# Supplementary figures and images for: Malignant Myoepithelioma of the Head and Neck: Demographics, Clinicopathological Characteristics, Treatment, and Prognosis
Source: Front Oncol. 2022 Jun 30;12:754967. doi: 10.3389/fonc.2022.754967 (PMC9279609; doi:10.3389/fonc.2022.754967)

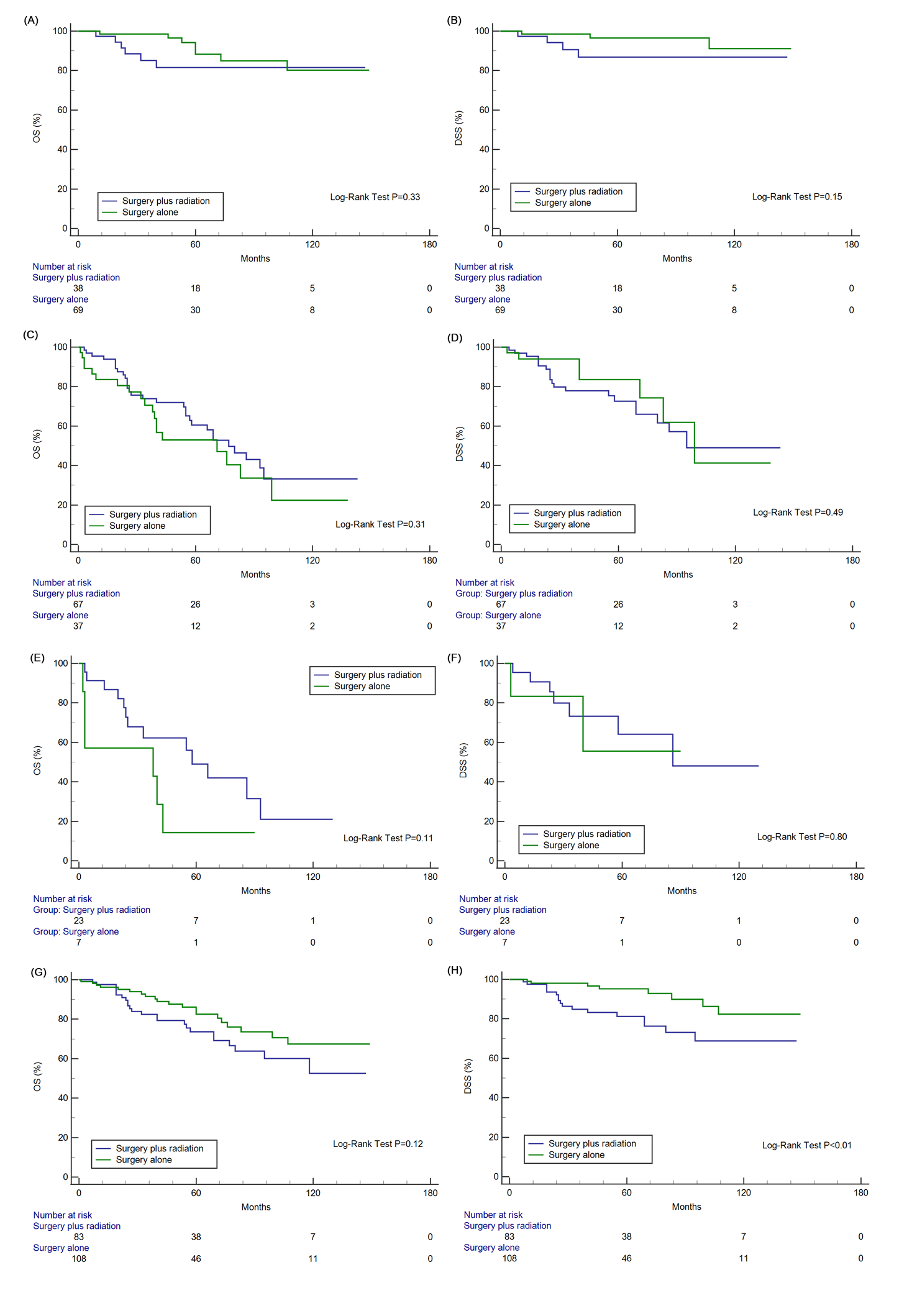

Supplement: Supplementary Figure 1 — Survival analysis stratified by treatment modalities (surgery plus radiation vs surgery alone) (A: TNM-I/II, OS; (B: TNM-I/II, DSS; (C: TNM-III/IV, OS; (D: TNM-III/IV, DSS; (E: N1+N2, OS; (F: N1+N2, DSS; (G: N0, OS; (H: N0, DSS). [file Image_1.tif]
