# Supplementary material for: Malignant Myoepithelioma of the Head and Neck: Demographics, Clinicopathological Characteristics, Treatment, and Prognosis
Source: Front Oncol. 2022 Jun 30;12:754967. doi: 10.3389/fonc.2022.754967 (PMC9279609; doi:10.3389/fonc.2022.754967)
Supplement: Supplementary file 2 [file DataSheet_1.docx]

Supplementary Table 1: Characteristics between patients with surgery plus radiotherapy and patients with surgery alone

| **Characteristics** | **Surgery plus radiotherapy (N=145)** | **Surgery Alone**  **(N=154)** | **P value** |
| --- | --- | --- | --- |
| Age (Year) |  |  |  |
| Mean | 62.0 | 59.5 | 0.19 |
| Median | 65 | 60 |  |
| Min | 18 | 20 |  |
| Max | 89 | 92 |  |
| Sex |  |  |  |
| Female | 68 | 80 | 0.42 |
| Male | 77 | 74 |  |
| Race |  |  |  |
| White | 105 | 112 |  |
| Black | 25 | 26 |  |
| Other (American Indian/AK Native, Asian/Pacific Islander) | 15 | 15 | 0.99 |
| Unknown | 0 | 1 |  |
| Tumor Grade |  |  |  |
| Well | 12 | 28 | <0.01 |
| Moderately | 34 | 37 |  |
| Poorly | 22 | 7 |  |
| Undifferentiated | 20 | 12 |  |
| Unknown | 57 | 70 |  |
| Primary Site |  |  |  |
| Salivary Gland | 108 | 118 | 0.07 |
| Oral Cavity | 18 | 25 |  |
| Nasal cavity & accessory sinuses | 10 | 10 |  |
| Pharynx & Larynx | 6 | 0 |  |
| Other | 3 | 1 |  |
| TNM |  |  |  |
| I | 14 | 34 | <0.01 |
| II | 24 | 35 |  |
| III | 34 | 21 |  |
| IV | 33 | 16 |  |
| Unknown | 40 | 48 |  |
| T category |  |  |  |
| T1 | 15 | 34 | <0.01 |
| T2 | 27 | 39 |  |
| T3 | 38 | 26 |  |
| T4 | 26 | 10 |  |
| Unknown | 39 | 45 |  |
| N category |  |  |  |
| N0 | 83 | 108 | <0.01 |
| N1 | 11 | 2 |  |
| N2 | 12 | 5 |  |
| Unknown | 39 | 39 |  |
| M category |  |  |  |
| M0 | 101 | 111 | 0.99 |
| M1 | 5 | 5 |  |
| Unknown | 39 | 38 |  |

Supplementary Table 2: Head-to-head Kaplan-Meier analysis of treatment modality

| Comparison | HR with 95%CI for OS | HR with 95%CI for DSS |
| --- | --- | --- |
| Surgery plus radiotherapy vs surgery alone | 1.39 (0.94-2.01) | **2.11 (1.22-3.64)** |
| Surgery plus radiotherapy vs radiotherapy alone | 0.33 (0.09-1.21) | 0.59 (0.10-3.38) |
| surgery alone vs radiotherapy alone | **0.24 (0.07-0.86)** | 0.28 (0.05-1.59) |
